# Nanotechnology Lighting the Way for Gene Therapy in Ophthalmopathy: From Opportunities toward Applications

**DOI:** 10.3390/molecules28083500

**Published:** 2023-04-15

**Authors:** Weiming Ren, Suyang Duan, Chao Dai, Chunbao Xie, Lingxi Jiang, Yi Shi

**Affiliations:** 1 Sichuan Provincial Key Laboratory for Human Disease Gene Study and Department of Laboratory Medicine, Sichuan Provincial People’s Hospital, University of Electronic Science and Technology of China, Chengdu 610072, China; 2 Health Management Center, Sichuan Provincial People’s Hospital, University of Electronic Science and Technology of China, Chengdu 610072, China; 3 Department of Laboratory Medicine, Sichuan Provincial People’s Hospital, University of Electronic Science and Technology of China, Chengdu 610072, China; 4 Research Unit for Blindness Prevention of Chinese Academy of Medical Sciences (2019RU026), Sichuan Academy of Medical Sciences & Sichuan Provincial People’s Hospital, Chengdu 610072, China; 5 Department of Ophthalmology, Sichuan Provincial People’s Hospital, University of Electronic Science and Technology of China, Chengdu 610072, China

**Keywords:** ophthalmopathy, nanocarrier, gene therapy, nucleic acid drug, delivery carriers

## Abstract

Hereditary ophthalmopathy is a well-described threat to human visual health affecting millions of people. Gene therapy for ophthalmopathy has received widespread attention with the increasing understanding of pathogenic genes. Effective and safe delivery of accurate nucleic acid drugs (NADs) is the core of gene therapy. Efficient nanodelivery and nanomodification technologies, appropriate targeted genes, and the choice of drug injection methods are the guiding lights of gene therapy. Compared with traditional drugs, NADs can specifically change the expression of specific genes or restore the normal function of mutant genes. Nanodelivery carriers can improve targeting and nanomodification can improve the stability of NADs. Therefore, NADs, which can fundamentally solve pathogeny, hold great promise in the treatment of ophthalmopathy. This paper reviews the limitations of ocular disease treatment, discusses the classification of NADs in ophthalmology, reveals the delivery strategies of NADs to improve bioavailability, targeting, and stability, and summarizes the mechanisms of NADs in ophthalmopathy.

## 1. Introduction

The incidence of ophthalmopathy is extremely high [1]. Visual impairment and hereditary ophthalmopathy have become major causes of disability, seriously affecting patients’ physical and mental health. According to World Health Organization (WHO) statistics, in 2019 about 2.2 billion people faced visual impairment or even blindness worldwide, of which at least 1 billion had not been treated (http://apps.who.int/iris/, accessed on 1 January 2023). Meanwhile, clinical ocular medications had some unignorable disadvantages, such as inefficient delivery, a small disease range, and poor patient compliance [2,3]. For some diseases, few effective treatments can help patients, such as age-related macular degeneration (AMD) [4], retinal pigmentation (RP) [5], choroid neovascularization (CNV) [6], Leber’s congenital amaurosis (LCA) [7], retinopathy of prematurity (ROP) [8], and Stargardt disease (STGD) [9].

Notably, nucleic acid drugs (NADs) use small nucleic acid to change the expression of target proteins through gene silencing that can be implemented to treat diseases [10]. The eye is one of the organs that benefit maximally from gene therapy [11]. The development of ophthalmology has gradually led to identifying genetic factors for hereditary ophthalmopathy. Various NADs can be designed to treat diseases according to different genetic factors [12]. In addition, NADs are simple to synthesize compared to antibody drugs and greatly broaden the drug target for ophthalmopathy [13]. In clinical trials, ocular tissue has been shown to have an isolated environment in the body [14]. Gene therapy using NADs can avoid significant immune response and systemic side effects. Therefore, gene therapy based on nucleic acid drugs has received widespread attention in treating ophthalmopathy.

In recent years, the rapid development of nanotechnology has provided huge help in gene therapy of ophthalmopathy, and there is an important opportunity for its application in clinical treatment. In this review, we focus on the classification and delivery carriers of NADs in treating ophthalmopathy and discuss the mechanism of NADs.

## 2. Classifications and Injection Schemes of NADs in Gene Therapy for Ophthalmopathy

### 2.1. Common NAD Types in Gene Therapy for Ophthalmopathy

**Figure 1:**
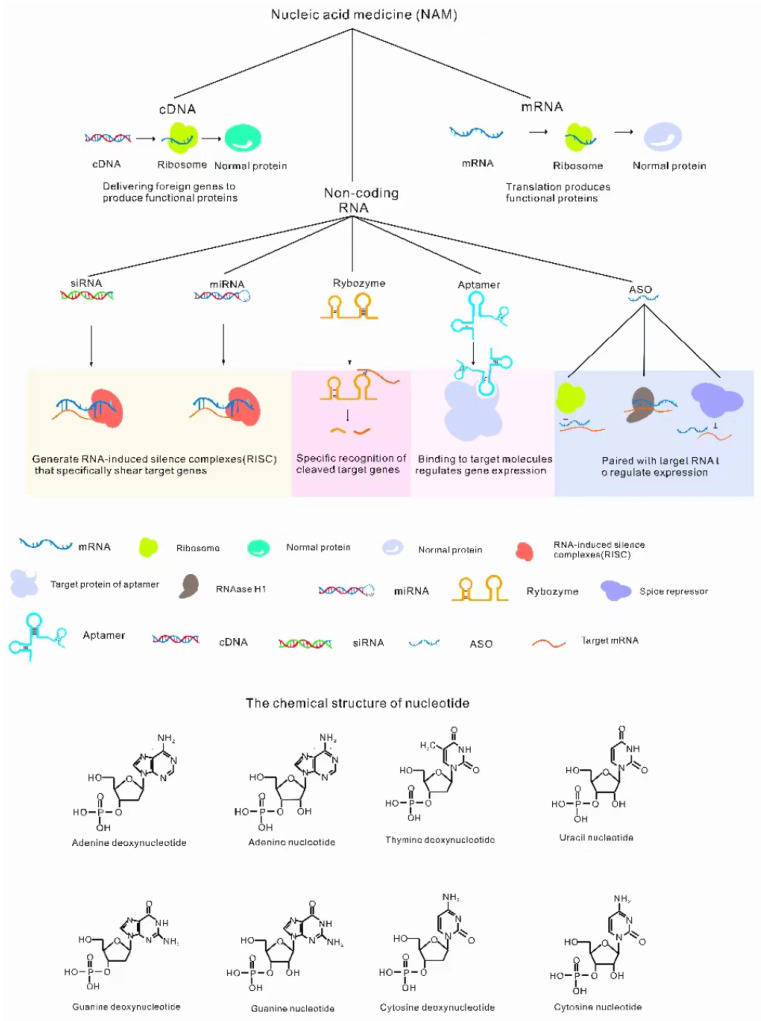
Classification of NADs. NADs include cDNA (translation function protein) [16], mRNA (translation function protein) [17] and noncoding RNAs [18]. Noncoding RNAs include ASO (antisense oligonucleic acid, binding target genes to regulate gene expression) [19], ribozymes (specific splicing RNA) [20], siRNA (RNA-induced silencing complexes (RISCs) degrade RNA) [21], miRNA (RISCs degrade RNA) [22], aptamers (specific binding to target molecules regulates gene expression) [23].

Gene therapy for ocular disease is usually accomplished by injecting carriers with different NADs into specific parts of the eyes [12]. NADs always refer to naturally occurring nucleotides or chemically modified nucleotides that can be used for the treatment of diseases, and are divided into cDNA, mRNA and noncoding RNA oligonucleotide drugs, as shown in Figure 1 [15]. Each type of NAD has different characteristics. The advantages, disadvantages and application preferences of common NADs in ocular treatment are shown in Table 1.

### 2.2. Gene Selection Options of NADs in Gene Therapy for Ophthalmopathy

Gene therapy will gradually become the mainstream means to treat common genetic diseases, and NADs are the core content. As we all know, NADs are primarily used for gene therapy; therefore, the selection of pathogenic genes is critical. At present, the selection of gene targets in ocular NADs is increasing, focusing on different eye diseases, such as retinal dystrophy, choroidopathy, and so on [28]. We review some reported disease-causing genes of ocular disease in Table 2.

### 2.3. Diverse Delivery Strategies Overcoming Delivery Efficiency in Ophthalmopathy

**Figure 2:**
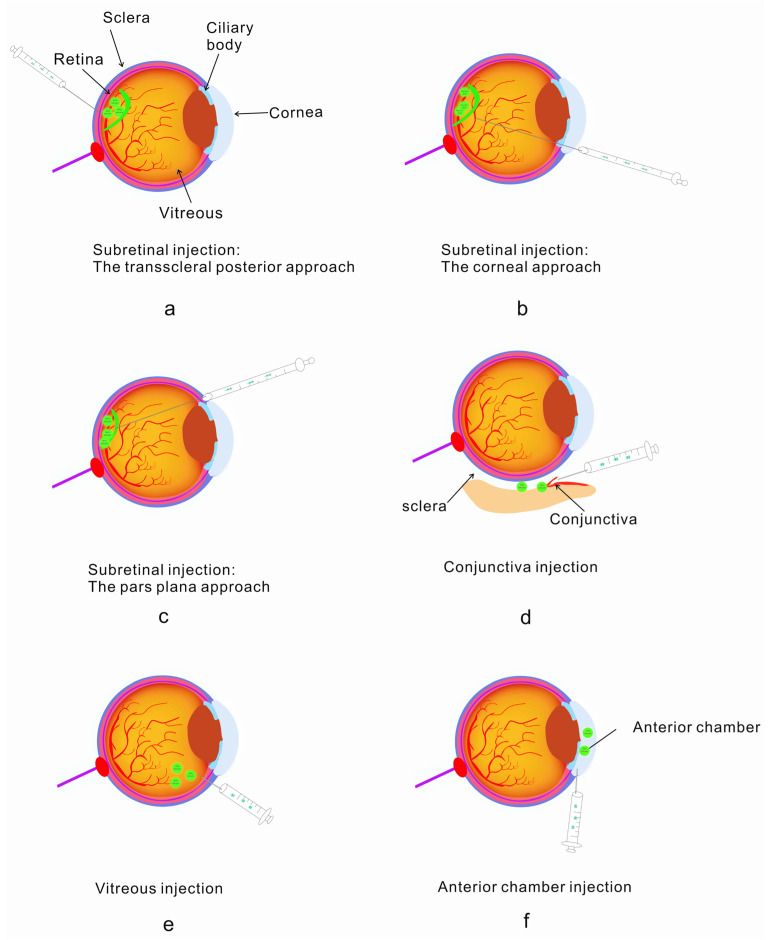
Standard implantable administration modes: subretinal injection, conjunctival injection, vitreous injection and anterior chamber injection [56,57]. (**a**) Retroscleral route—injected into the retina; (**b**) corneal injection into the subretina; (**c**) conjunctival injection using the pars plana route into the retina; (**d**) conjunctival injection; (**e**) vitreous injection; (**f**) anterior chamber injection.

A number of delivery protocols have been applied to the delivery of drugs. In the treatment of ophthalmopathy, common drug injection protocols are shown in Figure 2. NADs are widely used in the treatment of eye diseases, so the injection protocol is relatively mature. It has a huge market prospect. Currently, vitreous injection and subretinal injection are commonly used to treat neovascularization and retinal diseases. There are also a number of other injections depending on the disease, such as conjunctiva injection and anterior chamber injection (Figure 2).

## 3. The Application of Nanotechnology in Gene Therapy Can Help Improve Treatment

A key question in ocular disease gene therapy using NADs is whether these drugs can be delivered safely and efficiently to target cells in vivo. Several challenges must be overcome for the safe delivery of NADs to patients [58,59,60,61,62,63]: (1) low bioavailability of NADs; (2) poor targeting and drug distribution; (3) inadequate delivery options; and (4) unstable structures. To overcome the dilemmas, appropriate carriers and delivery methods are needed.

**Figure 3:**
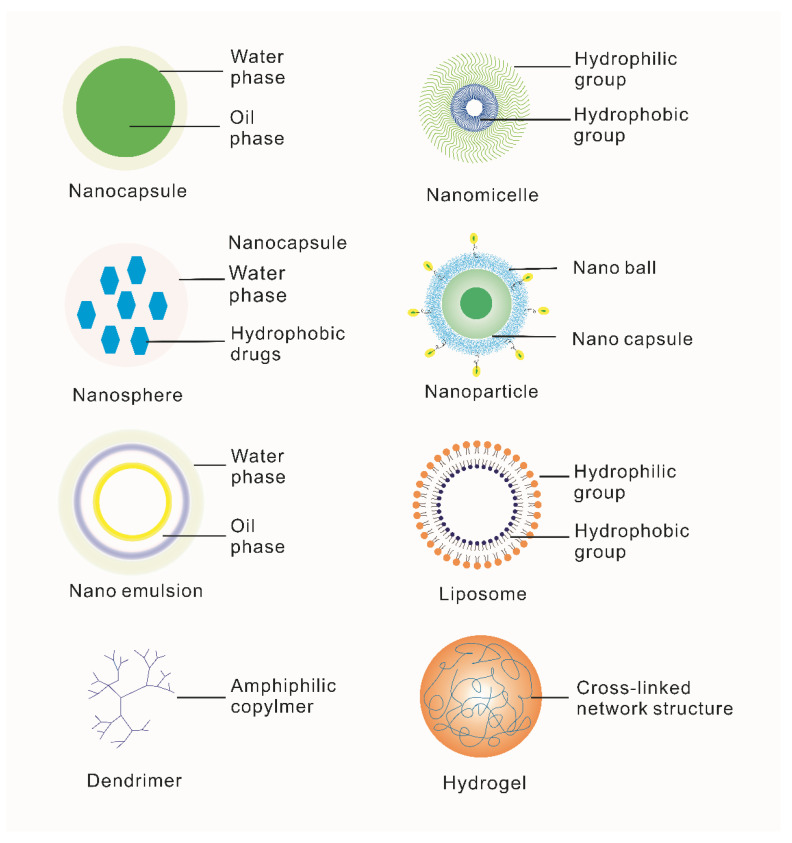
Classification and introduction of special biomimetic nanocarriers for ophthalmic diseases. Nanocapsules are nanoscale vesicle systems composed of nontoxic polymer membranes [65]. Nanomicelles are amphiphilic block copolymers [66]. Microspheres are tiny spherical entities formed by wrapping or adsorbing drugs in a polymer matrix [67]. Nanoparticles are granular dispersions or solid particles of size 10–1000 nm [68]. Nanoemulsions consist of oil, water, surfactant and cosurfactant [69]. Liposomes are artificial membranes. The hydrophilic head of phospholipid molecules is inserted into water and the hydrophobic tail of the liposome extends to the air. After agitation, the spherical liposome of double-lipid molecules is formed [70]. Dendrimers are a class of macromolecules with a highly branched structure obtained by the stepwise repetitive reaction of dendrimer elements [71]. Hydrogel is a kind of hydrophilic three-dimensional network structure gel [72].

It has been found that there are two main solutions to solve the above difficulties: either selecting suitable carriers for NAD delivery or improving the stability of NADs themselves. The emergence of nanotechnology has greatly contributed to implementing these two solutions [58]. First, conventional nanocarriers synthesized by nanotechnology are composed of natural or synthetic polymer materials such as nanomicelles, nanocapsules, liposomes, dendrimers, hydrogels, nanoparticles, nanoemulsion nanosphere (shown in Figure 3), which are widely reported to improve delivery efficiency. Secondly, the advent of DNA origami and other technologies has provided a broader application possibility for gene therapy [64].

### 3.1. NAD Carriers in Gene Therapy for Eye Diseases

As mentioned above, the two major factors limiting NAD therapy are the efficiency and safety of NAD delivery. The current paper focuses primarily on efficiently and accurately delivering NADs to target cells and minimizing safety concerns.

The eye has a unique physiological structure and protective mechanisms, such as the ocular blood–aqueous humor and the blood–retina physiological barriers that protect it from external infestations [73]. However, this also results in low bioavailability of the drug after the ocular administration of the carrier. Safe, accurate, and rapid administration of NADs to the eye is a central problem during the treatment of ocular disease. Currently, NAD delivery carriers that are commonly used in clinical and basic research can be divided into viral and nonviral delivery carriers (Table 3 and Table 4).

**Table 3:** Common viral carriers of NADs in ophthalmopathy.

Carrier	Advantages	Disadvantages	Target Tissue	References
Lentiviral carrier	Wide range of target cells and strong ability to carry foreign genes	Carcinogenic risk	Retinal ganglion cells, lens epithelial cells, corneal epithelial cells	[74,75,76]
Adenovirus carrier	Short expression time, High expression level of foreign genes	Strong immunogenicity	Photoreceptor cells, retinal pigment epithelial cells	[77]
Adeno-associated virus carrier	High infection efficiency	Strong immunogenicity	Photoreceptor cells, retinal pigment epithelial cells, retinal ganglion cells, lens epithelial cells	[78,79]

Lentiviral, adenoviral, and adeno-associated viral carriers are among the most common [74,75,76,77,78,79]. The advantages of viral carriers include high infection efficiency, wide host range, the simple structure of the viral genome, and ease of modification. Schnabolk et al. validated an alternative pathway inhibitor—a fusion protein consisting of complement receptor two fragments linked to the inhibitory domain of fH (CR2-fH)—as an efficacious treatment for choroidal neovascularization (CNV) when delivered intravenously [80]. The author constructed an AAV5 serotype containing an RPE-specific VMD2 promoter, which was targeted to RPE cells and expressed specifically. They tested an alternative approach of AAV-mediated delivery (AAV5-VMD2-CR2-fH or AAV5-VMD2-mCherry) using subretinal delivery in C57BL/6J mice. In summary, they found a significant reduction in the severity of ocular lesions following subretinal injection of AAV5-VMD2-CR2-fH in mice. The CR2-fH protein expressed by 3 × 10^8^ of the viral genome μg/mL AAV5-VMD2-CR2-fH in the RPE/choroidal CNV samples was similar to the level of purified CR2-fH protein at a therapeutic dose that was injected into the tail vein of the animal [81]. Viral carriers also have drawbacks, such as limited exogenous gene length and immunogenicity.

The nonviral carriers mainly include cationic lipid carriers, cationic polymers, lipid nanoparticles, inorganic nanoparticles, and biomimetic carriers. The advantages of nonviral carriers are the safety and sustained release of NADs. The main disadvantage is low transfection efficiency [81,82,83,84,85,86]. The nonviral carriers commonly used in NADs are currently applied in the treatment of ophthalmopathy, as shown in Table 4. Peeters et al. designed a kind of lipoplex (LPX) that could diffuse through the vitreous before reaching the retina with vitreous injection [82]. He modified the surfaces of LPXs with hydrophilic PEG chains, which prevented LPXs from aggregating in the vitreous. As he reported, the modified LPXs could freely move in the vitreous to overcome the vitreous barrier, which delivered NADs to the retina effectively. Luo et al. showed that a single intravenous injection of targeted, biodegradable nanoparticles delivering a recombinant Flt23k interceptor plasmid home to neovascular lesions in the retina regresses CNV in primate and murine AMD models [87], which offered a nanoparticle-based platform for targeted, vitreous-sparing, extended-release, nonviral gene therapy. Compared with invasive treatment, the delivery system reduced such risks as bleeding, pain, infection, and retinal detachment, and provided long-term inhibition of angiogenesis. As a result, more and more NAD carriers based on nanotechnology have been used to treat vascular eye diseases with subretinal and vitreous injections, and there are a lot of people working on related products [81,82,83,84,85,86].

**Table 4:** Common nonviral carriers of NADs in ophthalmopathy.

Nonviral Carrier	Advantages	Disadvantages	Target Tissue	References
Cationic lipid carrier	Increasing the local retention time of drugs, slow release of drugs, improving the bioavailability of drugs	Low transfection efficiency	Cornea, bulbar conjunctiva, sclera, retina,	[81,82]
Cationic polymer	Beneficial to endocytosis and will not be degraded by enzymes	High cytotoxicity	Retina	[83]
Lipid nanoparticles	High sustained release, high stability and low toxicity	Low transfection efficiency and hard to store	Cornea, retina	[84,85]
Inorganic nanoparticles	Easy to decorate, versatile	Poor biocompatibility	Cornea, retina	[86]

### 3.2. Nanocarriers Can Improve the Targeting and Biological Distribution of NADs in the Eyes

The bioavailability of NADs is poor due to the lack of suitable carriers [88]. The development of nanocarriers has been rapid in recent years, providing a new perspective for the treatment of ophthalmopathy by achieving controlled delivery, ensuring low irritation to the eye, improving drug bioavailability, or improving ocular histocompatibility. Various nanosystems have been designed to deliver the NAD payload to specific portions of the eye [89].

Many studies are exploring the applicability of all the above special biomimetic nanocarriers for ophthalmic diseases. For example, Cai et al. used subretinal injection to treat RP in young mice [90]. DNA nanoparticles are novel and specific, consisting of single molecules of DNA compacted with polyethylene glycol (PEG)-substituted lysine 30-mer peptides (CK30PEG10K), which can deliver a wide range of cargo sizes. One of the most common photoreceptor genes associated with RP is RDS, encoding the retinal degeneration slow (RDS) protein (also called peripherin/rds), which is a prime candidate gene for RP gene therapy [90]. The therapeutic gene (the normal mouse peripherin/rds) was carried by nanoparticles. They reported that a compacted DNA nanoparticle-mediated gene was delivered into the subretinal space of a juvenile mouse model of retinitis pigmentosa. The results showed that the transgenic products of nanoparticles were almost exclusively located in the outer cone segment of photoreceptor cells, which showed that the nanoparticles had good targeting. Furthermore, the cone function of the experimental group was significantly improved, and nanoparticles did not induce an acute immune response in the eye, which indicated that the nanoparticles were potential delivery vectors for gene therapy.

Jiang et al. encapsulated chitosan nanoparticles inside cationic lipid shells to design cationic lipid-like lipid particles (DLCS-NP) and demonstrated that the transporter targets conjunctival epithelial cells [91]. As they reported, the advantages of their nanocarrier are nucleic acid protection, excellent cellular uptake efficiency, use of multiple endocytic pathways, and the ability to escape endolysosomal transfer. It could play a role in drug delivery for gene therapy of ocular disease. This study transfected human conjunctival epithelial cells with the enhanced green fluorescent protein (EGFP) gene encapsulated by DLCS-NP and transfected human and rabbit conjunctival epithelial cells. DLCS-NP improved the expression of EGFP in both human conjunctival epithelial cells and rabbits. It is interesting to note that the specifically constructed cationic transporters increased the availability of NADs.

In conclusion, nanocarriers protect the NADs from degradation in vivo. In addition, nanocarriers can be modified to improve safety and targeting. Nanocarriers can also be permitted to deliver large fragment NADs because of the large payload.

### 3.3. Using Nanotechnology to Improve the Stability of NADs to Overcome Drug Instability

Most drugs in NADs can be chemically modified to increase stability, taking ribozymes as an example. When ribozymes are used as drugs, they can be engineered to meet specific needs during gene therapy [92]. Modifications to the widely used hammerhead and hairpin ribozymes are primarily based on conformation, mechanism of catalysis, and structural modification. Hendry et al. engineered the helix II length and size of the catalytic domain of hammerhead ribozymes and investigated the cleavage efficiency of a series of derivatives of the hammerhead ribozyme on a 550 nt segment of RNA in vitro [93]. Replacement of helix II with a G-C base pair enhances ribozyme activity. In hammerhead ribozymes [94], some nucleotide units in the loop II region were replaced with nucleotide units, such as the 2′-amino, fluorine, hydrogen and methoxy groups. The 3′-terminus was phosphorothioate-modified, as a result of which the stability of a ribozyme in human serum was increased 103-fold, and the efficiency of cleavage was increased 2-fold. Another example is DNA origami, which allows a long single-stranded DNA to be folded and self-assembled into a more stable nanostructure through the principle of base complementary pairing in hundreds or thousands of short-stranded DNA. Such efficient preparation of long single-stranded DNA sequences may also provide support for the development of new technologies such as gene tapping based on long-range single-stranded DNA fragments and long-range sequencing based on long-range locking probes. Although it has not been reported in ophthalmopathy, its advantages, such as biocompatibility, targeting, and stability, have wide application potential in ophthalmopathy. In general, different modifications of NADs can protect them from being degraded and thus improve stability. Simultaneously, appropriate modification can improve the efficiency of NADs.

Gene therapy based on NADs can be a good treatment for ophthalmopathy, and the application of nanotechnology further raises the possibility of treatment, so this is an important opportunity for future research. However, its clinical application requires a full understanding of the core therapeutic principles of NADs. Here, we introduce the mechanisms of different NADs.

## 4. The Mechanisms and Advantages of NADs in Gene Therapy for Ophthalmopathy

### 4.1. Principles and Advantages of Using cDNA as Therapeutic Nucleic Acid

Gene replacement therapy introduces normal wild-type genes into genetically defective cells to restore normal cellular function, usually with cDNA, which has been investigated in a variety of ophthalmopathy. The gene replacement regimen is primarily used to treat irreversible local retinal damage. A case in point is congenital Leber amaurosis (LCA) [95]. Retinal dystrophy is a genetically heterogeneous inherited retinal dystrophy that presents as blindness or severe visual impairment during childhood. Mutations in the *RPGRIP* gene have a strong association with LCA, and the gene encodes a photoreceptor protein to link retinitis pigmentosa GTPase regulator (RPGR) to the cilia [96]. Pawlyk et al. constructed the recombinant adeno-associated viral carrier AAV-mOps-RPGRIP-expressing normal RPGRIP [97]. As they reported, AAv-mediated RPGRIP gene replacement preserves photoreceptor structure and function in LCA mouse models, which showed gene replacement therapy may be effective in patients with LCA due to defects in RPGRIP and suggests the need for further preclinical development of gene therapy for this disease.

In ophthalmopathy with gene mutations such as LCA, cDNA may be an excellent choice to treat them because of stable expression in the patient’s genome and fewer adverse effects in patients.

### 4.2. Principles and Advantages of Using Small Interfering RNA (siRNA) as Therapeutic Nucleic Acid

**Figure 4:**
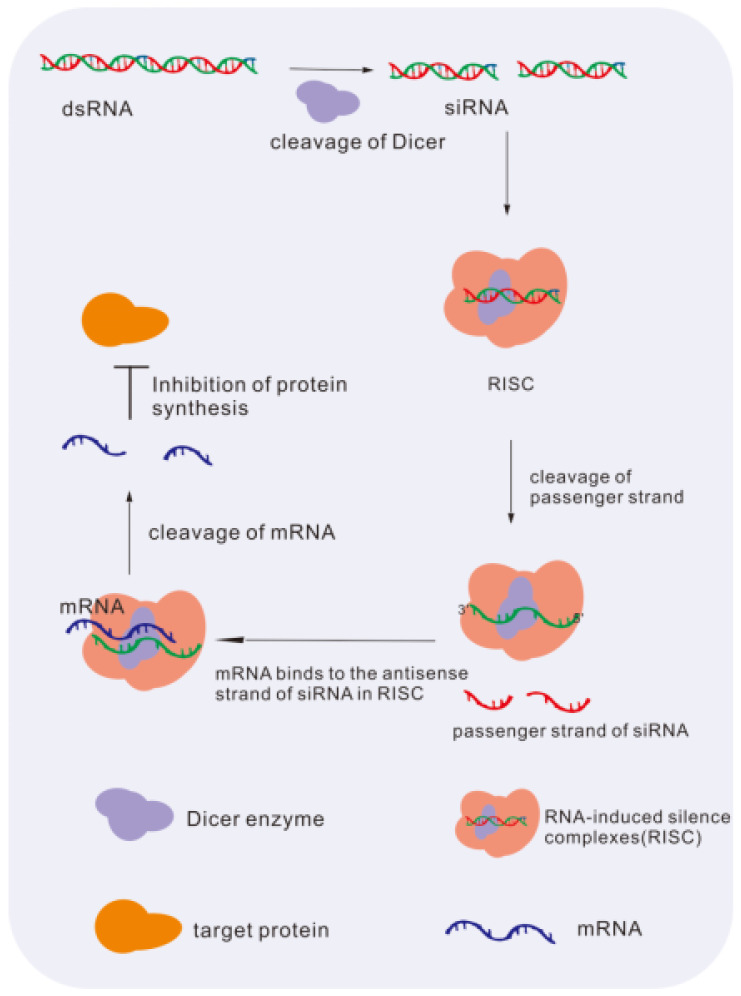
The mechanism of siRNA downregulates levels of gene. dsRNA is cleaved by the dicer enzyme to siRNA. siRNA merges into the RISC complex and binds to target mRNA for degradation. At last, the synthesis of protein is inhibited [98].

siRNAs are nucleotide duplexes of approximately 20 bp in length that can specifically couple and guide target gene degradation in cells, modifying the related signaling pathway for therapeutic intervention, and an example is shown in Figure 4 [98]. In ophthalmology, this gene interference therapy of NAD is considered one of the most useful and widely implemented in hereditary retinopathy.

siRNA-based therapy is a hot topic in the therapy of ocular disease [99]. As Li et al. reported, uveal melanoma (UM) is the most common and aggressive intraocular tumor in adults, with a high mortality rate, frequent recurrence, early involvement of local lymph nodes, and subsequent metastasis [100]. Chemotherapy drugs such as cisplatin are chosen to treat UM [101], but they produce toxic and adverse side effects on normal tissue and UM may have resistance to drugs. Li reported that lncRNA OUM1 was overexpressed in UM, functions as a catalyst and regulates protein tyrosine phosphatase (PTP) activity by binding to PTP receptor type Z1 (PTPRZ1), which played an important role in cell proliferation, metastasis and chemotherapy resistance in the UM microenvironment. Thus, they designed siRNAs that could selectively knock down the lncRNA *OUM1 (siOUM1*) and its target gene *PTPRZ1* (*siPTPRZ1*) to inhibit the activity of PTP. As a result, the UM proliferation and metastasis were suppressed and the cisplatin sensitivity in UM cells was improved. In the subcutaneous xenograft model and pulmonary metastasis model, knocking down *OUM1* and *PTPRZ1* showed good tumor inhibition efficiency.

siRNA has high efficacy in knocking down the expression of both target genes and pathogen genes to achieve the therapeutic effect, and it can be used with small-molecule or antibody drugs to improve the efficacy of treatment [98]. In addition, the siRNA can be designed widely by the nucleic acid sequence identity of ocular disease pathogen genes. At the same time, the synthesis of therapeutic siRNA is easy [99].

### 4.3. Principles and Advantages of Using microRNA (miRNA) as Therapeutic Nucleic Acid

miRNAs is a short noncoding RNAs consisting of 19–23 nucleotides, which inhibits transformation and protein synthesis at the mRNA level. Therefore, miRNA is considered to be involved in many life activities [102], such as gene expression and regulation, cellular differentiation and injury. miRNA has also been widely proven to play an important role in retinal development and injury, and is likely to be a significant breakthrough to treat retinal disease.

For example, ROP is defined as preterm infants less than 36 weeks of age with low birth weight and prolonged inhalation of oxygen [103]. In their nonvascularized retina, fibroangioma proliferates and contracts and further causes traction retinal detachment and blindness. Among all treatments, vascular endothelial growth factor (VEGF) can significantly promote new blood vessel formation in ROP pathogenesis [104]. VEGF is a highly specific provascular endothelial cell growth factor that promotes increased vascular permeability, extracellular matrix degeneration, vascular endothelial cell migration, proliferation and angiogenesis. It includes many family members, such as VEGF-A, VEGF-B, and VEGF-C, which play a pivotal role in the pathogenesis of AMD [30]. The treatment of AMD usually depends on combined surgical and medical treatment therapy. Injecting anti-vascular endothelial growth factor (anti-VEGF) can effectively control the growth of VEGF, and is considered a good method to maintain patients’ existing vision in surgical treatment therapy [105]. However, anti-VEGF has been reported to have highly individual specificity and is expensive for patients [103]. Currently, the commonly used treatment is the vitreous injection of VEGF antibodies. However, such a treatment scheme cannot avoid the high rate of late recurrence. Therefore, many studies hope to use miRNA to knock down *VEGF* expression for the treatment of neovascular disease.

In a previous study [106], miR-126 exhibited the effect of *VEGF-A* suppression in RPE cell lines that were conserved across species, while other miRNAs showed inconsistent effects. Intravitreal delivery of miR-126 effectively downregulated intraocular *VEGFA* expression and further significantly reduced pathological retinal neovascularization in a rat model of induced retinopathy (OIR). This study demonstrated that vitreous injection of miR-126 into rats negatively regulates *VEGFA* expression and inhibits retinal neovascularization formation, demonstrating promising therapeutic potential for miR-126 in the treatment of ROP.

Because there are already a large number of basic and clinical research seats, the use of miRNA as an NAD is a relatively mature scheme for the treatment of ophthalmopathy. It has obvious advantages. For example, miRNAs regulate the expression of endogenous genes at the posttranscriptional level and play an important role in the development of various types of ophthalmopathy, so miRNAs have wide therapeutic potential in ophthalmopathy [107].

### 4.4. Principles and Advantages of Using mRNA as Therapeutic Nucleic Acid

Using mRNA as therapeutic nucleic acid is novel. In comparison to other classes of NADs, such as plasmid DNA, mRNA can be translated directly into proteins in the cytoplasm without entering the nucleus to be transcribed and will not be inserted into the genome in order to cause insertion mutations to occur [108]. Gene editing technology can be used to knock down *VEGF* in AMD. Therefore, ocular drug studies at the mRNA level have received increasing attention in recent years.

A special lentiviral carrier system was constructed by Ling, S et al. to combine CmRNA with lentiviral particles to form the lentiviral particle-bearing messenger RNA (mLP) [109]. They added Cas9 mRNA to the middle of the mLP, and then inserted the gDNA into the U3 region at the 3′ end of the long lentivirus region. Compared to conventional Cas 9, mlp Cas9 has a higher knockout efficiency of the mouse *VEGF* gene and a shorter duration of presence, which can safely and efficiently complement AMD therapy.

mRNA has received much attention in terms of tumors, vaccines, rare diseases and other directions. Its application in treating ocular disease has not yet been reported, but mRNA-based drugs have great potential. These drugs can be translated directly into the cytoplasm to generate specific proteins for treatment with a more rapid duration of action than DNA and siRNA drug [110]. Furthermore, mRNA drugs do not enter or alter the host genome. Advances in mRNA drug research have led to significant attention to the application of miRNAs in ocular disease.

### 4.5. Principles and Advantages of Using Aptamer as Therapeutic Nucleic Acid

The aptamer is an oligonucleotide that binds with high affinity and specificity to a variety of target molecules, such as DNA, RNA and peptides [111]. Aptamer specifically binds to target molecules to affect their function. The aptamer can be used in the treatment of ocular disease. Carrasquillo et al. combined nanodelivery technology with aptamers to develop a controlled drug delivery system for long-term inhibition of vascular endothelial growth factor (VEGF) and its mediated response, which could be used to treat diseases of the choroid and retina [112]. As they reported, they used poly(lactic-co-glycolic) acid (PLGA) microspheres to contain anti-VEGF RNA aptamer (EYE001) formulations. It spread in the sclera and inhibited VEGF activity, which shows its potential in the treatment of choroid and retinal diseases.

### 4.6. Principles and Advantages of Using Ribozymes as Therapeutic Nucleic Acid

Ribozymes can specifically cleave RNA molecules to reduce gene expression. Each ribozyme contains a substrate-binding and catalytic structure. The former binds to a specific RNA via complementary base pairing and the latter catalysis RNA strand breakage [113].

Notably, Liu et al. successfully constructed an RP disease model using AAV-mediated ribozyme and knocked down the mRNA expressing cGMP phosphodiesterase (PDEγ) in rods in the retina of wild-type mice, thereby generating a retinal degeneration animal model [114]. The advantage of the ribozyme is that it can efficiently knock down wild-type *PDEγ* mRNA in vivo, reducing the target RNA and leading to a loss in rod photoreceptors and in rod-mediated ERG amplitudes, thus generating an animal model of retinal degeneration resembling human RP in an essentially normal adult retina. In addition, this carrier ribozyme technique could be applicable to other genes associated with RP and perhaps also to mRNAs of phototransduction genes not yet associated with RP.

## 5. Clinical Transformation and Application

Some NADs have advanced to clinical trials after basic research as well as a variety of delivery carriers, as shown in Table 5. Recently, the clinical transformation and research of cDNA, siRNA, ASO, and aptamers have been popular in gene therapy for ophthalmopathy.

cDNA was usually used to replace the wrong gene and recover eye function. As reported by Jacobson et al., LCA can be treated with an in vitro subretinal injection of the *RPE65* gene [115]. They recruited patients for clinical trials., and 15 patients’ visual function improved after receiving subretinal injections of *rAAV2-hRPE65*. The results showed that the cDNA drug was effective and could be used in follow-up trails.

siRNA drugs were also used in clinical studies to decrease wrong protein expression and worked well. For example, AGN211745^TM^ is a siRNA drug that can treat AMD effectively [116]. Vision and fovea thickness were improved in AMD patients after a single intravitreal injection of AGN211745. This siRNA drug was also well tolerated in phase 2 clinical trials.

ASO is another popular NAD in clinical trials to bind and inhibit the function of target RNA. In recent years, one ASO drug, ISTH0036, was used to treat glaucoma. It is an antisense oligonucleotide against TGF-β2 [117]. To assess its safety, a phase 1 clinical trial was conducted. Results showed that ISTH0036 was safe when injected intravitreally at the end of glaucoma filtration surgery.

The aptamer has been used to bind to target molecules that affect functions in the treatment of ocular disease. Pegaptanib sodium (Macugen^®^; Pfizer Inc., New York, NY, USA) was an aptamer drug developed by Pfizer for treating AMD that has been approved for commercial release by the FDA [118]. As Patricia reported, pegaptanib sodium was also effective in a retinal vascular disorder named branch retinal vein occlusion (BRVO) [119]. In a clinical trial, five eyes of five patients were treated with intravitreal injection of pegaptanib sodium. Patients showed significant improvement in visual acuity and macular edema after 3 months, indicating that pegaptanib sodium was safe and efficacious in the treatment of BRVO.

Therefore, the use frequency and success rate of NADs that rely on nanotechnology improvement in different eye diseases are very high.

**Table 5:** NAD clinical trials.

Drugs	Target Gene	Delivery System	Disease Type	Status	Clinical Trials Gov Identifier	Reference
rAAV2-CBSB-hRPE65(cDNA)	RPE65	Recombinant adeno-associated virus serotype 2 (rAAV2)	Amaurosis of Leber	Phase 1 active, not recruiting	NCT00481546	[7]
rAAV2-VMD2 (cDNA)	MERTK	rAAV2	Retinitis pigmentosa	Phase 1 completed	NCT01482195	[120]
RGX-314 (cDNA)	VEGF	rAAV2	Neovascular AMD degeneration	Phase 1	NCT03066258	[121]
Pegaptanib (aptamer)	VEGF	Carrier-free	Wet AMD degeneration	Phase 3	NCT01189461	[119]
vMCO-I (cDNA)	MCO	rAAV2	Retinal degeneration	Phase 1/2	NCT04919473	[122]
QR-1123 (ASO)	P23H	Water-based formulation	Retinal dystrophies	Phase 1/2	NCT04123626	[123]
SYL040012 (siRNA)	ADRB2	Carrier-free	Glaucoma	Phase 1	NCT00990743	[124]
SYL1001 (siRNA)	TRPV1	Carrier-free	Dry-eye disease	Phase 3	NCT03108664	[125]
AGN211745 (siRNA)	VEGF-1	Carrier-free	Neovascular AMD	Phase 1/2	NCT00363714	[116]
ISTH0036 (ASO)	TGF-β2	Water-based formulation	Primary open-angle glaucoma	Phase 1	NCT02406833	[117]

## 6. Future Perspectives

There are limitations to traditional medicine in treating ophthalmopathy, and only parts of ophthalmopathy may be entirely cured [28]. A range of potential genetic drivers of eye disease have been identified in recent years [126,127]. Therefore, an increasing number of ocular gene therapies are being progressively used in clinical practice [128]. Through many clinical trails on different phase, one of the most widely used gene therapies, NAD, is considered to have the greatest potential of curing ophthalmopathy [129]. The clinical transformation of NADs is also in full swing, especially the clinical transformation of cDNA drugs, siRNA drugs, ASO and aptamers in retinal diseases. There are a number of NADs in phase 1/2 clinical trials targeting different ophthalmopathy, as mentioned above.

At the same time, limitations and challenges for NADs have come to the forefront in clinical translational studies. Firstly, NADs drugs lack targeting and suitable distribution in eyes [130,131]. Nanotechnology can be used to create suitable carriers to help these limitations and challenges [89,132,133]. In addition, NADs are immunogenic, which may trigger the immune response and reduce security, so NADs need to be modified to reduce immunogenicity when used [134]. At the same time, delivery carriers such as viral vectors often have high immunogenicity. Therefore, nonviral vectors with low immunogenicity, especially nanocarriers created by nanotechnology, have received extensive attention and research [135]. What is more, the stability of NADs is also a problem that must be solved in clinical transformation. For different NADs, the design of nanocarriers based on nanotechnology can improve their stability in vivo. Preclinical and clinical trial design of NADs also requires careful consideration. For example, preclinical carcinogenicity of drugs, selection of disease models, treatment effects, treatment factors and research objects in clinical trials are all problems that need to be solved in the clinical transformation of NADs [136].

Although most nanocarriers delivering NADs are still in the basic research stage, there are many clinical trials reported on nanocarriers delivering conventional drugs to treat ophthalmopathy, which provided clinical research experience to deliver NADs. For example, the FDA approved a clear water nanobeam formulation named OTX-101 0.09% (CEQUA™; cyclosporine A 0.09%) in 2018 to increase tear production in patients with keratoconjunctivitis sicca. The drug uses nanomicelles to wrap hydrophobic cyclosporine A (CsA) within its hydrophilic core, which helps CsA better disperse and dissolve into the precorneal tear membrane when dropped into the patient’s eye [137]. A novel ocular drug delivery technology based on cyclodextrin nanoparticles for diabetic macular edema has entered phase 2/3 clinical trials. Preclinical and clinical work has demonstrated that cyclodextrin nanoparticle eye drops effectively deliver drugs to the back of the eye, increasing absorption and reducing systemic distribution of drugs, thereby reducing side effects [138].

Nanotechnology has many advantages, but its clinical application still has some limitations. Firstly, the safety of nanomaterials with carrier functions and nanomaterials engineered as NADs by nanotechnology is one of the issues limiting their application. Some nanomaterials are still toxic when used. In addition, the biodegradability of nanomaterials is also questionable. Lastly, the high cost of current nanotechnology-based NADs may limit their use in future markets [139]. However, with the progress of nanotechnology, the development of new materials, optimization of the preparation process and so on, these problems will be gradually solved.

Gene therapy is the hope of patients with severe ophthalmopathy. However, in past years, these limitations have made many ophthalmopathy types untreatable. With improvements in carriers and NADs with nanotechnology, gene therapy for ophthalmopathy is more promising, and the success rate and safety are gradually improving. Therefore, nanotechnology lights the way for gene therapy in ophthalmopathy.

## Figures and Tables

**Table 1:** Four different types of common NADs in ophthalmopathy.

Types	Characteristics	Advantages	Disadvantages	Examples	References
cDNA	Long nucleotide sequences encoding specific proteins	High stability	Possibly inserted genome	rAAV8-Reep6.1 (rescuing Reep6 mutation via gene replacement therapy)	[24]
siRNA	Short nucleic acid double strand	Specific knockdown gene expression	Poor stability	AGN211745^TM^ (Treatment of AMD, phase 2 clinical trial)	[25]
Antisense oligonucleotide	Hairpin structure	Precise regulation of gene expression	Poor stability	ISTH0036 (Treatment of glaucoma, phase 1 clinical trial)	[26]
Aptamers	Oligonucleotides specifically binding to DNA, RNA, proteins	Simple synthesis, low cost and wide range of action targets	Screening difficulty	ARC1905^TM^ (Phase 1 clinical trial of combination therapy with Lucentis^®^ 0.5 mg/eye for neovascular AMD)	[27]

**Table 2:** Identified Disease-causing Genes In ophthalmopathy.

Diseases	Disease-Causing Gene	Expression Location	References
Neovascular glaucoma (NVG)	*GNAQ*, *CFH*, *Y402H*	Retina	[29,30]
Age-related macular degeneration (AMD)	*ARMS2/HTRA1*, *Y402H*, *ARMS2*, *VEGF-R*, *EST1*	Choriocapillaris	[31,32,33,34]
High myopia (HM)	*ZNF644*, *P4HA2*, *SLC39A5*, *BSG*, *LRPAP1*, *LEPREL1*, *CTSH*, *OPN1*, *LW*, *ARR3*	Retina, retinal pigment epithelium	[35,36,37,38]
Retinitis pigmentosa (RP)	*RHO*, *PRPF31*, *USH2A*, *Peripherin/RDS*, *NRL*, *RP1*, *RGR*, *ABCA4*, *RPE65*, *CNCG*	Retina	[39,40,41,42]
Primary congenital glaucoma (PCG)	*CYP1B1*, *MYOC*, *LTBP2*, *FOXC1*	Cornea, ciliary body, iris and retina	[43,44,45,46]
Congenital aniridia	*PAX6*	Lens, iris	[47]
Xerophthalmia	*TRP*	Cornea	[48]
Cataract	*CRYAA*, *COL4A1*, *BFSP*	Lens	[49,50,51]
Uveitis	*Peripherin/RDS*, *DRB1/DQA1*, *IL23R/C1orf141 ADO/ZNF365/EGR2*	Iris, lens, choroid	[52]
Choroidal neovascularization (CNV)	*SDF-1*, *CXCR4*, *VEGF*	Choriocapillaris	[53]
Diabetic retinopathy (DR)	*VEGF*, *AR*, *AGE*, *RAGE*, *ACE*, *NOS*	Vitreous vessels	[54,55]

## Data Availability

The data presented in this study are available upon request from the corresponding author.
